# A Technique for Performing Electrical Impedance Myography in the Mouse Hind Limb: Data in Normal and ALS SOD1 G93A Animals

**DOI:** 10.1371/journal.pone.0045004

**Published:** 2012-09-28

**Authors:** Jia Li, Wayne L. Staats, Andrew Spieker, Minhee Sung, Seward B. Rutkove

**Affiliations:** 1 The Department of Neurology, Harvard Medical School, Beth Israel Deaconess Medical Center, Boston, Massachusetts, United States of America; 2 The Department of Mechanical Engineering, Massachusetts Institute of Technology, Boston, Massachusetts, United States of America; University of Florida, United States of America

## Abstract

**Objective:**

To test a method for performing electrical impedance myography (EIM) in the mouse hind limb for the assessment of disease status in neuromuscular disease models.

**Methods:**

An impedance measuring device consisting of a frame with electrodes embedded within an acrylic head was developed. The head was rotatable such that data longitudinal and transverse to the major muscle fiber direction could be obtained. EIM measurements were made with this device on 16 healthy mice and 14 amyotrophic lateral sclerosis (ALS) animals. Repeatability was assessed in both groups.

**Results:**

The technique was easy to perform and provided good repeatability in both healthy and ALS animals, with intra-session repeatability (mean ± SEM) of 5% ±1% and 12% ±2%, respectively. Significant differences between healthy and ALS animals were also identified (e.g., longitudinal mean 50 kHz phase was 18±0.6° for the healthy animals and 14±1.0° for the ALS animals, p = 0.0025).

**Conclusions:**

With this simple device, the EIM data obtained is highly repeatable and can differentiate healthy from ALS animals.

**Significance:**

EIM can now be applied to mouse models of neuromuscular disease to assess disease status and the effects of therapy.

## Introduction

Electrical impedance myography (EIM) is a technique in which a high-frequency, low-intensity electrical current is applied via surface electrodes to discrete areas of muscle and the resulting voltages measured [1]. From these, the muscle’s electrical characteristics, including its resistance, reactance, and phase are calculated. Studies have shown the potential value of these EIM values as biomarkers for assessing disease status and progression, in amyotrophic lateral sclerosis [2], spinal muscular atrophy [3], and myositis [4]; ongoing work suggests that it could also be potentially useful as a diagnostic tool in helping to differentiate neuromuscular illness [5].

In addition to human studies, EIM has also been applied to rat disease models [6]–[8]; such studies have been aimed at developing a deeper knowledge of the relationship between EIM data and underlying pathology. Perhaps more importantly, it can be used as a tool to quickly evaluate drug efficacy during pre-clinical drug development. In the rat, the technique has been performed on the gastrocnemius muscle using a straightforward modification of the human technique in which 4 discrete adhesive electrodes are placed in a line on the skin overlying the muscle following the long axis of the limb [6]. Excellent reproducibility can be achieved using this simple approach.

For a variety of reasons, however, it would useful to be able to study mouse neuromuscular disease models as well. Indeed, there is an ever-increasing number of mouse models of neuromuscular disease and a convenient, easily-applied, tool that does not require animal sacrifice could find wide application. However, configuring EIM for use in the mouse hind limb is not straightforward since the mouse limb is considerably smaller than that of the rat (2 cm circumference at mid calf for the mouse versus 6 cm for the rat) and it is not possible simply to mimic the approach used in human subjects using still smaller adhesive electrodes as was done in the rats. Moreover, it would be advantageous to also measure electrical current flow in multiple directions, so as to assess the muscle’s electrical anisotropy. Such measurements may provide additional insight into muscle condition, including potentially differentiating neurogenic from myopathic disease [5]. Although such measurements were attempted previously using small metal strips [9], the technique was very difficult to perform given the mouse’s size.

Thus, in this study, we describe a new methodology for performing EIM on the adult mouse hind limb using a fixed rigid electrode array and provide results in both healthy animals and those with ALS.

**Figure 1:**
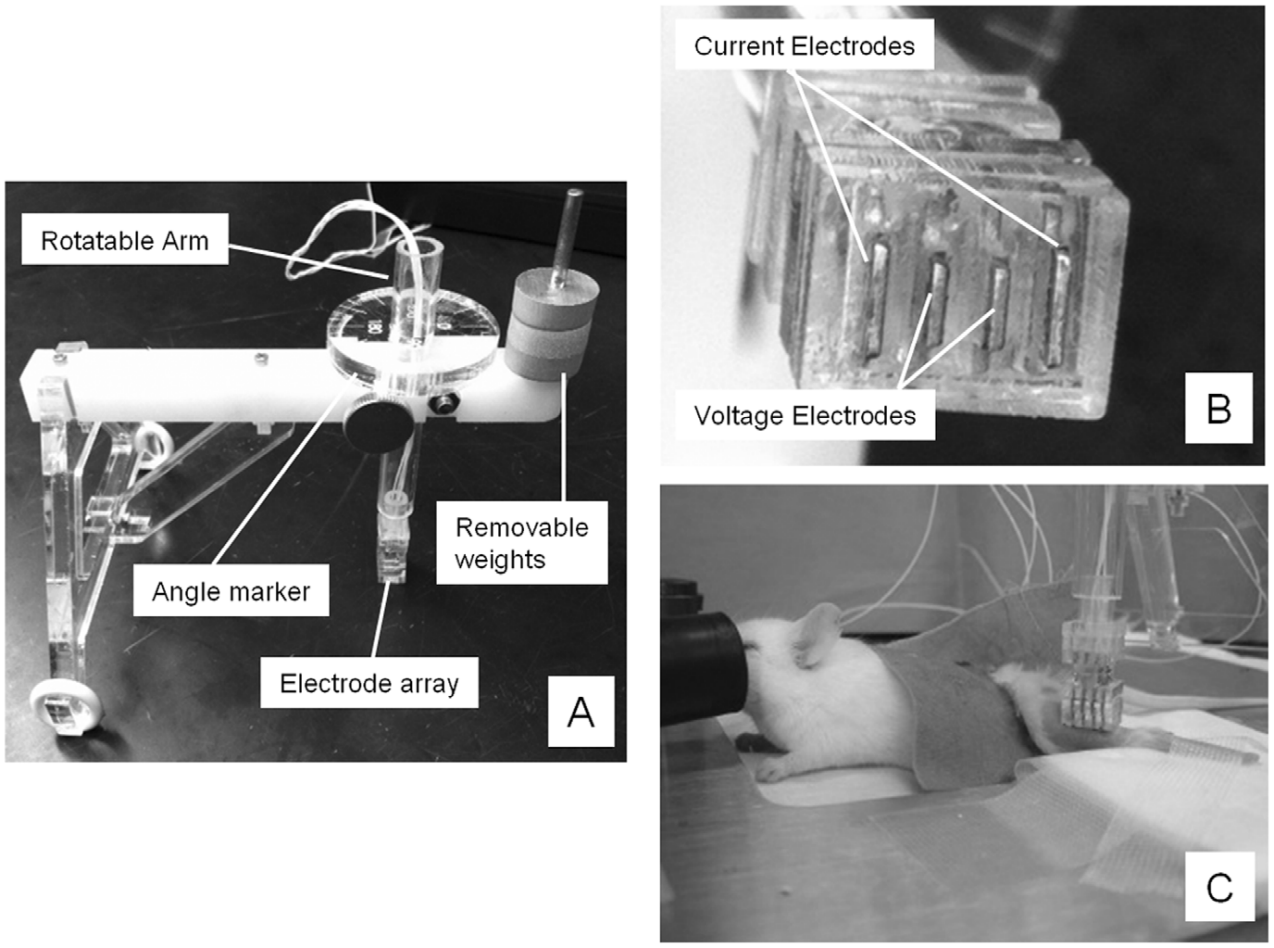
Multiple views of the device. A. The entire device, showing the rotatable arm used to change the direction of the electrode array, the angle marker, and the removable weights; B. A close-up view of the tetrapolar electrode array; C. the entire animal setup with the device overlying the gastrocnemius muscle.

## Methods

### The Electrode Array

Figure 1 shows three different views of the device. Four stainless steel strips placed in parallel were embedded within an acrylic head, with each of the individual strips protruding slightly to help ensure good contact with the skin. In particular, the electrode head was an assembly of 5 flat laser-cut acrylic pieces and 4 flat water-jet-cut stainless steel plates. The stainless steel plates were cut into a stepped shape so they could be encased perpendicularly in the stack of acrylic pieces, with their location entirely determined by the location of mating slots in the acrylic pieces. The stainless steel plates also had a zigzag pattern of notches around which a copper wire was wound; the sharp edges of the notches ensured good electrical contact with the copper wire, which connected to the measurement equipment. The stainless steel plates were positioned in the acrylic stack and permanently encased by adhering the acrylic pieces together. Finally, the end of the stainless steel plates that protruded slightly to serve as the contacting portion of the electrode head was filed to remove any burrs or sharp edges. The total footprint of the array was set at 3.95 by 6.85 mm, a dimension based on measurements performed on the hind limbs of multiple adult animals,. The two outer strips (to serve as current-emitting electrodes) were 0.55 mm wide and 3.95 mm long; the two inner strips (to serve as voltage-measuring electrodes) were 0.55 mm wide and 2.85 mm long. The strips were 0.55 mm apart (measured from the center of each strip).

**Figure 2:**
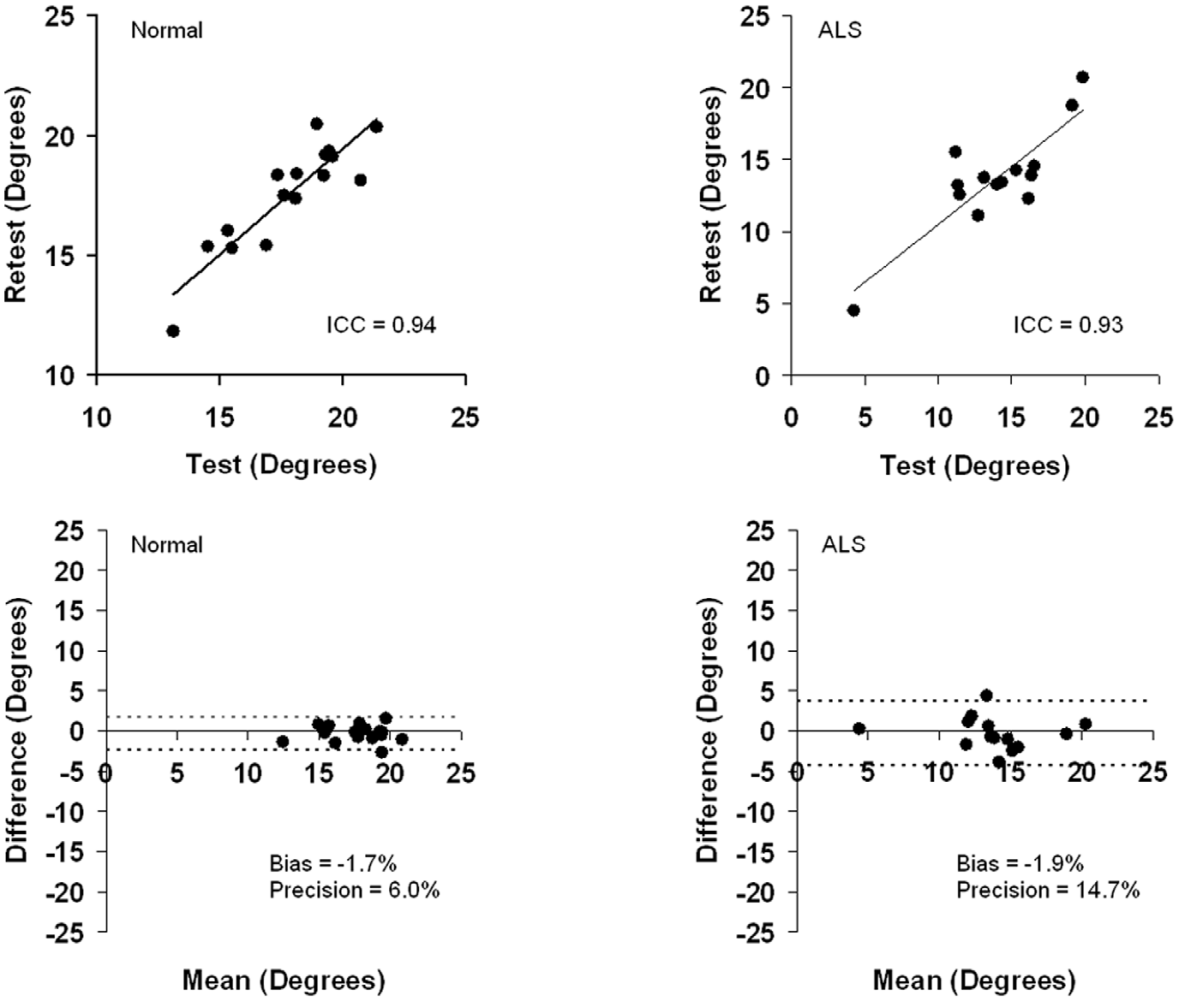
The test-retest and Bland-Altman plots of phase at 50 kHz for healthy and ALS mice in the longitudinal configuration. ICC values are 0.94 for healthy mice and 0.93 for ALS mice.

**Table 1:** Repeatability Analyses at 50 kHz.

		Resistance (Ω)		Reactance (Ω)		Phase (°)	
		Long	Trans	Long	Trans	Long	Trans
**Normal**	**ICC**	0.90	0.88	0.95	0.89	0.94	0.88
	**Diff% ± SEM**	5% ±1%	6% ±1%	7% ±2%	10% ±2%	5% ±1%	7% ±2%
	**Bias**	4.0%	4.0%	2.2%	3.7%	−1.7%	−0.1%
	**Precision**	5.0%	8.1%	8.7%	12.5%	6.0%	9.5%
**ALS**	**ICC**	0.76	0.79	0.83	0.84	0.93	0.88
	**Diff% ± SEM**	9% ±2%	8% ±2%	17% ±4%	14% ±4%	12% ±2%	11% ±3%
	**Bias**	−1.2%	1.3%	−1.7%	−1.0%	−1.9%	−1.5%
	**Precision**	12.4%	9.2%	24.6%	19.4%	14.7%	13.3%

Long, longitudinal; trans, transverse.

The head of the device was affixed to a rotatable arm that could be placed squarely over the animal’s gastrocnemius muscle. The rotatable arm included an embedded plastic disc with angle markers allowing the array to be consistently placed relative to the long axis of the limb. The electrode wires extended through the center and out the top of the rotatable arm where they connected to an impedance-measuring device. In addition, a dowel pin extension parallel to the rotatable arm allowed for the addition of weights to ensure consistent contact pressure of the device against the muscle with each measurement.

**Figure 3:**
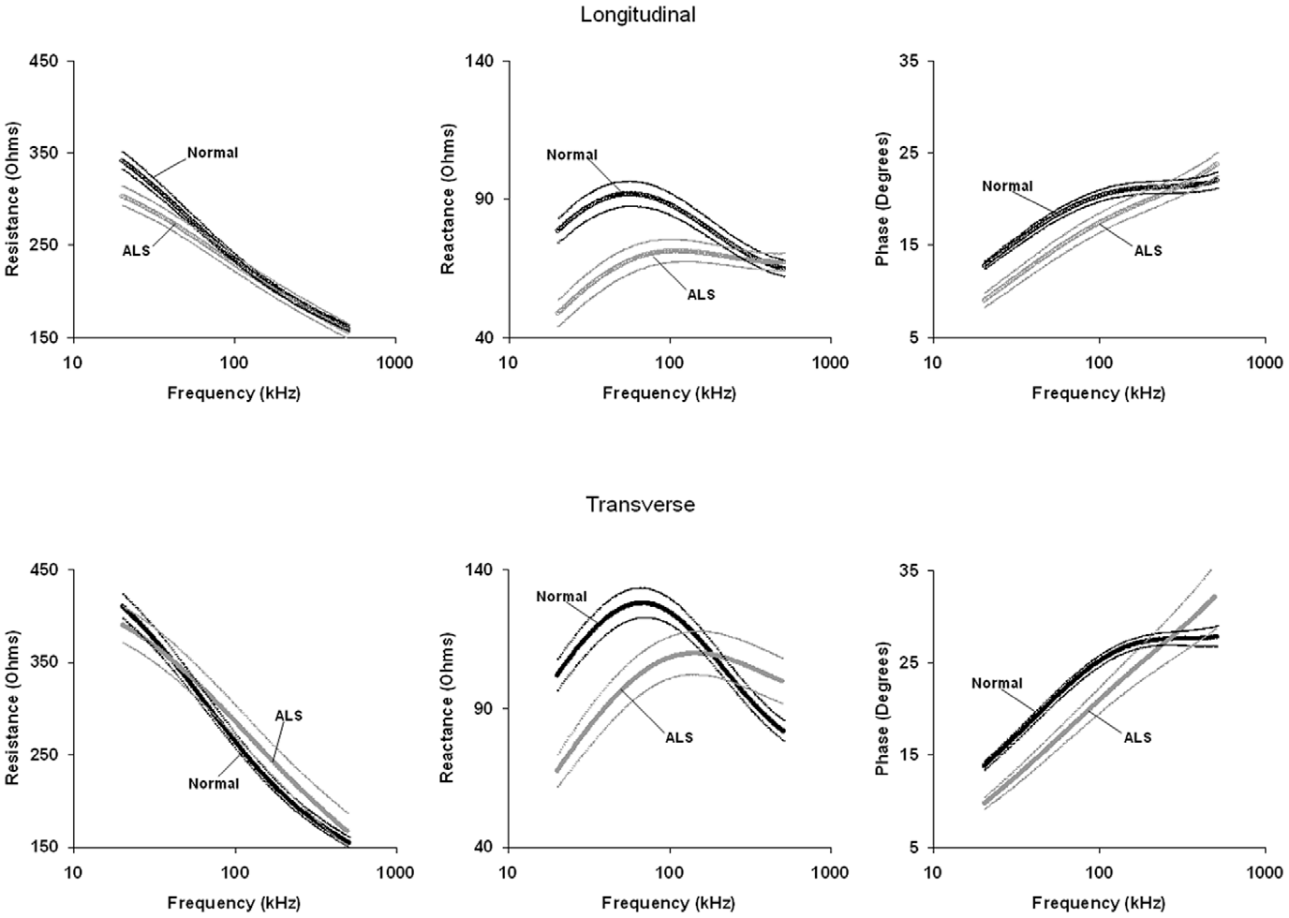
Major multifrequency EIM parameters (± SEM) for healthy and ALS mice in both longitudinal (0°) and transverse configurations (90°).

**Table 2:** Longitudinal and Transverse EIM Data in Normal and ALS Mice at Selected Frequencies.

Longitudinal						
		20 kHz	50 kHz	100 kHz	300 kHz	500 kHz
**Resistance (Ω)**	**Normal**	341±9.5	282±6.4	235±4.7	179±3.4	161±3.1
	**ALS**	303±10.6	265±8.9	229±8.6	178±8.4	157±8.1
	**p value**	**0.0112**	0.1191	0.5354	0.8931	0.6673
**Reactance (Ω)**	**Normal**	79±4.4	92±4.6	88±4.0	70±2.9	65±3.0
	**ALS**	49±4.8	66±4.9	71±4.1	68±3.1	67±3.0
	**p value**	**0.0001**	**0.0007**	**0.0067**	0.6117	0.6199
**Phase (°)**	**Normal**	13±0.4	18±0.6	20±0.6	21±0.7	22±0.9
	**ALS**	9±0.8	14±1.0	17±1.1	21±1.1	24±1.2
	**p value**	**0.0002**	**0.0025**	**0.0248**	0.9936	0.2787
						
**Transverse**						
		**20 kHz**	**50 kHz**	**100 kHz**	**300 kHz**	**500 kHz**
**Resistance (Ω)**	**Normal**	410±13.0	333±9.9	263±8.0	181±6.1	156±5.5
	**ALS**	390±19.7	338±17.8	285±16.9	201±17.8	167±19.2
	**p value**	0.3930	0.7649	0.2135	0.2270	0.5205
**Reactance (Ω)**	**Normal**	102±5.5	126±5.6	124±4.7	94±3.6	82±3.7
	**ALS**	68±5.7	96±7.4	108±7.5	105±8.3	100±8.2
	**p value**	**0.0003**	**0.0034**	0.0672	0.1903	**0.0410**
**Phase (°)**	**Normal**	14±0.5	21±0.6	25±0.6	28±0.8	28±1.1
	**ALS**	10±0.6	16±1.1	21±1.4	28±2.6	32±3.5
	**p value**	**<0.0001**	**0.0003**	**0.0049**	0.7187	0.1865

### Impedance Measurement System

EIM measurements were performed with the Imp SFB7® bioimpedance spectroscopy device (ImpediMed, San Diego, CA). This device could perform measurements over the 4 kHz to 1 MHz range within just several seconds. For the analyses that follow, however, only values from 20 kHz to 500 kHz were included.

**Figure 4:**
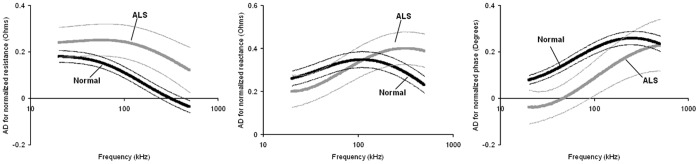
Normalized multifrequency anisotropy differences (± SEM) of all 3 major EIM parameters (resistance, reactance, phase) for healthy and ALS mice.

### Animals

8 male and 8 female healthy Swiss-Webster mice, obtained from Charles River Laboratories, of 18 weeks of age were used for the reproducibility studies. In addition, 7 male and 7 female ALS SOD1 G93A animals (strain B6SJL-Tg(SOD1*G93A)1Gur/J), bred from animals obtained from Jackson Laboratories were also studied at 18 weeks of age. Animals were housed 5 per cage with access to food *ad libitum*. All studies were approved by the Beth Israel Deaconess Medical Center Institutional Animal Care and Use Committee (IACUC).

### Animal Measurement Procedures

All EIM measurements were performed with the animals placed under 1% isoflurane anesthesia delivered by nosecone with a heating pad underneath the limb to maintain consistent temperature. After the fur was clipped, a depilatory agent was applied to the skin to remove all remaining fur; the skin was then cleaned with 0.9% saline solution. The leg was then taped to the measuring surface at an approximately 45 degree angle extending out from the body (Figure 1 C). To assist in repeat placement of electrodes, a pinpoint tattoo was placed close to the center of the gastrocnemius muscle at a point approximately 2/3 of the distance between the midpoint of the lumbar spine and the base of the heel pad of each mouse.

For assessment of electrical anisotropy (directional dependence of electrical current flow through the tissue), the electrode array was placed both parallel (i.e., 0° named longitudinal configuration) and perpendicularly (i.e., 90°named transverse configuration) to the major muscle fiber direction by rotating the device arm.

For assessment of technique repeatability, after an initial set of measurements was made, the animal was removed from the set-up, lifted up, and then replaced about 1–2 minutes later and new adhesive tape applied; the array was then placed on the leg and the measurements repeated.

### Data Analysis

For assessment of immediate repeatability of the testing system in all 16 healthy and 14 ALS mice, the percent variation between measurements, intra-class correlation (ICC) coefficients, and Bland-Altman analyses [10] were calculated. Comparisons between normal and ALS animals were made using unpaired t-test at selected frequencies. For all analyzes, significance was taken at p<0.05, two tailed. The three major impedance parameters included: the resistance, a measure of opposition to current flow; the reactance, a measure of the ability of the tissue to briefly store and release charge; and the phase, a ratio of these two basic impedance parameters calculated via the relationship phase  =  arctan (reactance/resistance). All three measures have demonstrated potential value in clinical studies [1].

## Results

### Reproducibility of the Technique in Healthy and Normal Mice at 50 kHz

Table 1 provides a summary of percent difference, intra-class correlation values and Bland-Altman analyses of EIM measurements at 50 kHz for both healthy and ALS mice at both longitudinal and transverse configurations. Figure 2 provides a graphical example of those data for phase values. As can be seen, excellent reproducibility was achieved with this technique. For example, for healthy mice, the percent difference of means (± SEM) between the first and second set of measurements for phase was just 5% ±1% in longitudinal configuration and just 7% ±2% in transverse configuration.

### Differences between Normal and ALS Animals

Figure 3 shows the multifrequency resistance, reactance, and phase data averaged across the group of 18-week-old healthy mice and the 18-week-old ALS mice in both longitudinal and transverse positions. For the transverse measurements, data was only obtained from 11 ALS mice since in 3 the atrophy was sufficiently severe such that the electrode array could not fit entirely over the muscle in the transverse direction. For both directions, resistance decreases at higher frequencies for both healthy and ALS mice, although the frequency dependence is greater in the healthy animal. Both reactance and phase show a peak in the healthy mouse; however the peaks are no longer present in the ALS mouse. These changes in the multifrequency spectrum are similar to those observed in human ALS [11] and in the previous rat [8]. Table 2 shows the data at selected frequencies with the associated p values. Consistent with those previous studies, the differences between healthy and diseased muscle was most apparent at frequencies below 100 kHz. Of note, the reactance showed much greater differences than the resistance at these frequencies. This finding is consistent with the reactance’s being highly dependent on the cumulative area of the muscle fiber membranes and the consequent charge storage capability. In contrast, at these frequencies, the resistance is mainly dependent on extracellular water content and is thus less sensitive to the muscle fiber atrophy.

The transverse-longitudinal differences can also be summarized by calculated a normalized anisotropy difference at each frequency. This is completed by subtracting the longitudinal from the transverse measurement at each frequency, divided by the mean value at that frequency. The resulting multifrequency curves for both the normal and ALS animals is plotted in Figure 4, which differ substantially, paralleling previously multifrequency anisotropy changes in seen in ALS patients [12].

## Discussion

In this study we have developed and tested a relatively simple device for performing EIM measurements on the calf of the mouse hind limb. The device provides relatively good intra-session reproducibility, both in healthy and in diseased animals, and can also differentiate between the groups, greatly improving upon an initial effort at obtaining these measurements [9]. Moreover, the device is capable of performing measurements at both 0° and 90° relative to the major muscle fiber direction.

The product used here was specifically designed and engineered for this study and is not commercially available; however, if having such a system were of sufficient general interest, it would be a straightforward matter to create similar versions using this basic design.

Although straightforward to perform, like any procedure, it did take some practice to become fluid and consistent with its application. It was critical to ensure that the array was sitting precisely at the same angle when performing the measurements and was clearly centered over the pinpoint tattoo. The moistening of the animal’s skin with saline solution also needed to be performed consistently by only applying a damp gauze pad repeatedly to the limb. Finally, repeated application of the array did cause irritation to the skin. Additional experience suggested that the procedure should not be performed more than twice weekly, lest irritation to the skin occur with secondary scabbing, likely related to the repeat placement of the array and use of the depilatory agent.

Several aspects of the results obtained here deserve further discussion. First, the repeatability in normal animals was somewhat better than that achieved in the advanced ALS animals. Although the reasons for this are uncertain, several factors may play a role including altered skin integrity in ALS [13], small limb size (making the electrode array more sensitive to small differences in placement), and lower impedance values for reactance and phase, making identical absolute variations in parameters larger when calculated as a percent. Second, the time between repeated measurements was short and could not be expected to account for other potential physiological variations to the measured impedance, such as those due to exercise or hydration status, that could contribute to reduced reproducibility in a real-world study. Third, this study was only intended to introduce and evaluate the reproducibility of the EIM technique in mice and was not meant to assess EIM’s ability to monitor ALS progression in the SOD1 mouse model. In order to study this fully, it would be important to compare EIM’s ability to monitor progression in mice with more standard approaches such as measurement of body weight, functional status, and motor unit number estimation. It does remain possible, however, that EIM could specifically serve as a potential replacement for the more challenging and time-consuming technique of motor unit number estimation. Indeed, the previous study in ALS rats suggested that EIM provided a much more easily obtained, effective surrogate measure for motor unit number estimation [8]. Whether this will also hold true in the ALS mouse models remains to be determined.

At the frequencies of electrical current being applied here, resistance mainly provides data on the extracellular milieu of the muscle. For example, increasing endomysial fat will increase the resistance values, whereas muscle edema would reduce it. The reactance, in contrast, is mainly impacted by sarcolemmal surface area and thus is especially sensitive to myocyte atrophy. Accordingly, reactance shows the greatest alteration in denervating disorders where atrophy is most extreme. Since the phase is a ratio of these two measures, it is a more non-specific parameter. However, phase appears to be very sensitive in a variety of contexts since myocyte atrophy is often accompanied by alterations in the endomysium.

As suggested by this explanation of the EIM parameters, unlike standard electromyography, in which the inherent electrical activity of the muscles and nerves is measured, in EIM it is more the histologic status of the tissue that is assessed. Accordingly, EMG is best suited for showing situations in which resting membrane potential stability and action potential morphology are being altered such as in the evaluation of myotonic conditions or nerve hyperexcitability. Standard needle EMG is also likely best for evaluating conditions in which only selected muscles are affected (e.g., due to an isolated nerve injury), since in EIM the flow of electrical current cannot be confined to a single muscle. In comparison, EIM is potentially useful in conditions in which the alterations are histological and membrane stability and function is thought to be stable, such as in disuse or muscle wasting, in which EMG is typically normal, and in myopathies in which EMG alterations can be very subtle. In most other conditions, however, both EMG and EIM can be used, each offering something different. For example, in ALS, EIM is very sensitive to alterations in disease progression [14], whereas EMG is sensitive to disease onset [15].

In summary, this approach provides a simple and reproducible method for performing EIM in the mouse. Given the current and expanding plethora of animal models of neuromuscular disease, the ability to obtain rapid data on muscle condition may prove valuable in a variety of contexts including assisting with the identification of disease onset, disease severity, and monitoring the effects of therapy. For example, it could serve a role in preclinical drug testing such that the effect of therapy of muscular dystrophy or motor neuron diseases could be assessed without animal sacrifice and without performing time-consuming and often inconsistent function tests. Moreover, this technology allows further exploration of the relationship between different types of muscle pathology and the surface impedance characteristics, making it possible to expand further the application of EIM to different disease models.
